# Symptomatic Human Hantavirus in the Americas

**DOI:** 10.3201/eid1302.061023

**Published:** 2007-02

**Authors:** Jan Clement, Guy H Neild, Piet Maes, Herwig Leirs, Patrick Matthys, Marc Van Ranst

**Affiliations:** * University of Leuven, Leuven, Belgium; † Royal Free Hospital, London, United Kingdom; ‡ University of Antwerp, Antwerp, Belgium

**To the Editor**: In a recent letter (*1*), dos Santos et al. described 3 cases of hantavirus pulmonary syndrome (HPS) from Juquitiba and stated that “the first human cases of symptomatic infection by hantaviruses were reported from Brazil in 1993.” However, we described 8 cases of symptomatic hemorrhagic fever with renal syndrome (HFRS) in Recife, Brazil, 5 months before the initial May 1993 report of Sin Nombre virus (SNV)–induced HPS in the United States (*2*). Our report was therefore the first published account of symptomatic hantavirus infections, not just in Brazil but anywhere in the Americas (*3*).

Serum samples from our Brazilian HFRS cases, collected in 1990, were screened by an immunofluorescence assay (IFA) and ELISA for immunoglobulin G, as were the current Brazilian HPS cases (*1*). Two of our patients had an increased immunoglobulin M titer by ELISA (*2*). Rat-transmitted Seoul virus (SEOV) was considered most likely because this was the only hantavirus strain showing clear positive results in IFA (*2*,*3*). All the Recife cases in 1990 had reported likely rat contacts and were initially diagnosed as leptospirosis with acute renal failure and thrombocytopenia, clinical hallmarks of both HFRS and leptospirosis (*3*). We also subsequently found evidence of SEOV infection in 31 (15%) of 201 leptospirosis-suspected acute renal failure cases from Belém, Brazil, confirmed in 1 case with highly specific neutralization tests (*4*). Moreover, as we predicted (*3*), some of the 133 (41%) of 326 urban cases of acute renal failure from Salvador, Brazil, which appeared nonconfirmed for leptospirosis (*5*), were later shown to be caused by SEOV (unpub. data). Finally, of 379 schoolchildren from Salvador at high risk for frequent rat exposure, 13.2% were IFA positive for the Korean prototype Hantaan virus (HTNV) but none for the American SNV (*6*). Because both HTNV and its rodent reservoir are absent from the American biotope, HTNV seroreactivity should be considered a cross-reaction to another related murine antigen; that is to say, the ratborne SEOV.

Wild rats (*Rattus rattus and R. norvegicus* ) are the only Old World rodents ubiquitous in the New World and thus a potential source of SEOV infection in the Americas (*3*,*7*). Moreover, the first hantavirus characterized in South America was SEOV, isolated as long ago as 1984 from a rat caught in Belém (7). Furthermore, the first 3 clinical cases of hantavirus infection in the United States were SEOV-induced (Baltimore rat virus) HFRS cases and not HPS (*8*).

The clinical syndromes of HFRS and HPS can appear identical, with pulmonary edema, shock, and renal insufficiency with marked proteinuria and thrombocytopenia (*9*). Moreover, worldwide ELISA testing with a single antigen such as SNV or Puumala virus (PUUV) can result in misleading cross-reactions, since both viruses are genetically related. Although this SNV-PUUV cross-reactivity enabled the first recognition of HPS cases in the New World 13 years ago, this may now lead to the wrong clinical diagnosis and reinforces the need for specific tests such as neutralization tests or reverse transcription–PCR. Although not as lethal and probably not so frequent as HPS, SEOV-induced HFRS may still be greatly underestimated in the Americas, or misdiagnosed as leptospirosis.

## References

[1] dos Santos MC, de Lacerda MV, Benedetti SM, Albuquerque BC, de Aguiar Filho AA, da Rosa Elkhoury M, Human hantavirus infection, Brazilian Amazon. Emerg Infect Dis. 2006;12:1165–7. 16845782 10.3201/eid1207.060074 PMC3291060

[2] Hinrichsen S, Medeiros de Andrade A, Clement J, Leirs H, McKenna P, Matthys P, Hantavirus infection in Brazilian patients from Recife with suspected leptospirosis. Lancet. 1993;341:50. 10.1016/0140-6736(93)92523-V 8093292

[3] Clement J, Neild G, Hinrichsen SL, Crescente JA, van Ranst M. Urban leptospirosis versus urban hantavirus infection in Brazil. Lancet. 1999;354:2003–4. 10.1016/S0140-6736(05)76782-9 10622333

[4] Clement J, Hinrichsen S, Crescente J, Bigaignon G, Yersin C, Muthusethupathi M, Hantavirus-induced hemorrhagic fever with renal syndrome (HFRS) has to be considered in the differential diagnosis of leptospirosis-suspected cases in the New and the Old World. Am J Trop Med Hyg. 1999;61(Suppl):316–7.

[5] Ko AI, Galvaõ Reis M, Ribeiro Dourado CM, Johnson WD Jr, Riley LW. Urban epidemic of severe leptospirosis in Brazil. Salvador Leptospirosis Study Group. Lancet. 1999;354:820–5. 10485724 10.1016/s0140-6736(99)80012-9

[6] Mascarenhas-Batista AV, da Rosa ES, Ksiazek TG, da Rosa AP, LeDuc JW, Pinheiro F, J. Anti-hantavirus antibodies in school children in Salvador, Bahia. Rev Soc Bras Med Trop. 1998;31:433–40. 10.1590/S0037-86821998000500003 9789441

[7] LeDuc JW, Smith GA, Pinheiro FP, Vasconcelos PF, Rosa ES, Maiztegui JI. Isolation of a Hantaan-related virus from Brazilian rats and serologic evidence of its widespread distribution in South America. Am J Trop Med Hyg. 1985;34:810–5. 2862803 10.4269/ajtmh.1985.34.810

[8] Glass GE, Watson AJ, LeDuc JW, Childs JE. Domestic cases of hemorrhagic fever with renal syndrome in the United States. Nephron. 1994;68:48–51. 10.1159/000188086 7991040

[9] Clement J, Colson P, McKenna P. Hantavirus pulmonary syndrome in New England and Europe. N Engl J Med. 1994;331:545–6. 10.1056/NEJM199408253310813 8041425

